# Chemical Composition and Preliminary Toxicity Evaluation of the Essential Oil from *Peperomia circinnata* Link var. *circinnata*. (*Piperaceae*) in *Artemia salina* Leach

**DOI:** 10.3390/molecules26237359

**Published:** 2021-12-03

**Authors:** Késsia do Socorro Miranda Mesquita, Bruna de Souza Feitosa, Jorddy Neves Cruz, Oberdan Oliveira Ferreira, Celeste de Jesus Pereira Franco, Márcia Moraes Cascaes, Mozaniel Santana de Oliveira, Eloisa Helena de Aguiar Andrade

**Affiliations:** 1 Faculdade de Farmácia, Universidade Federal do Pará, Rua Augusto Corrêa S/N, Guamá, Belém 66075-900, PA, Brazil; kessiakit@hotmail.com (K.d.S.M.M.); brunaufpa08@gmail.com (B.d.S.F.); eloisa@museu-goeldi.br (E.H.d.A.A.); 2 Laboratório Adolpho Ducke-Coordenação de Botânica, Museu Paraense Emílio Goeldi, Av. Perimetral, 1901, Terra Firme, Belém 66077-830, PA, Brazil; jorddynevescruz@gmail.com (J.N.C.); oberdan@museu-goeldi.br (O.O.F.); celeste.frango12@gmail.com (C.d.J.P.F.); 3 Programa de Pós-Graduação em Biodiversidade e Biotecnologia—Rede Bionorte, Universidade Federal do Pará, Rua Augusto Corrêa S/N, Guamá, Belém 66075-900, PA, Brazil; 4 Programa de Pós-Graduação em Química, Universidade Federal do Pará, Rua Augusto Corrêa S/N, Guamá, Belém 66075-900, PA, Brazil; cascaesmm@gmail.com

**Keywords:** natural products, volatile compounds, bioactive compounds, molecular docking

## Abstract

*Peperomia* Ruiz and Pav, the second largest genus of the Piperaceae, has over the years shown potential biological activities. In this sense, the present work aimed to carry out a seasonal and circadian study on the chemical composition of *Peperomia circinata* essential oils and aromas, as well as to evaluate the preliminary toxicity in *Artemia salina* Leach and carry out an in silico study on the interaction mechanism. The chemical composition was characterized by gas chromatography (GC/MS and GC-FID). In the seasonal study the essential oil yields had a variation of 1.2–7.9%, and in the circadian study the variation was 1.5–5.6%. The major compounds in the seasonal study were β-phellandrene and elemicin, in the circadian they were β-phellandrene and myrcene, and the aroma was characterized by the presence of β-phellandrene. The multivariate analysis showed that the period and time of collection influenced the essential oil and aroma chemical composition. The highest toxicity value was observed for the essential oil obtained from the dry material, collected in July with a value of 14.45 ± 0.25 μg·mL^−1^, the in silico study showed that the major compounds may be related to potential biological activity demonstrated by the present study.

## 1. Introduction

The Piperaceae family, characterized as basal angiosperms [1], has approximately 3600 thousand species distributed both in pantropical and neotropical regions [2]. Many species are described as presenting themselves as herbs, sub-shrubs, shrubs, or arbors, or lianas, epiphytes, rupicolous, or terrestrial [3]. Furthermore, this family is divided into five genera: *Macropiper*, *Zippelia*, *Piper*, *Peperomia,* and *Manekia* [4].

Peperomia Ruiz and Pav is the second largest genus of the Piperaceae family, containing about 1600 species [5], and is considered one of the 10 main genus rich in floristic plants species [6]. This genus species are endemic to the Amazon and the Andes, with distribution in tropical and subtropical regions around the world, although these species are more concentrated in the Americas, where there is the greatest habitat diversity, from the southern United States to Argentina and Chile [5]. In Brazil, there are around 162 species, mainly in the Atlantic Forest [7], being morphologically described as presenting opposite or verticillate leaves and webbed, pinnate, or sometimes obscured veins [8].

Many Piperaceae species are aromatic, promising in essential oils production [9], with potential biological activities, such as: antiprotozoal [10], larvicidal [11], psycho-neuropharmacological [12], antifungal [13], insecticidal [14], antibacterial [15], and toxicity [16]. Among these biological activities, it is important to mention that studies focused on essential oils’ toxicity have aroused great interest [17,18,19], due to their biological properties that contribute to the improvement and functioning of several products, especially in food [20], and for that, some studies use in the preliminary assessment of essential oils toxicity against *Artemia salina* Leach, which is a small crustacean, widely used in these tests, due to its practicality, speed, safety, and economy. In addition, the great importance of this test is related to the acetylcholinesterase (AChE) enzyme, which has been the target of *A. salina*, through which larvae mortality occurs when they come into contact with the essential oil in the presence of light, and these interactions may indicate a possible biological activity [21].

Regarding the essential oils’ chemical composition from *P. circinnata* Link var. *circinnata*, there are only two records in the literature [22,23]; however, in the essential oils from *Peperomia* genus, a variety of compounds classes can be found, such as: hydrocarbon and oxygenated monoterpenes, hydrocarbon and oxygenated sesquiterpenes, benzenoids, and phenylpropanoids [24], and the diversification in the essential oils’ chemical profile of this genus can be observed in the *Peperomia serpens* essential oil, which is characterized by the major compounds (*E*)-nerolidol (38.0%), ledol (27.1%), and α-humulene (11.5%) [25]. Conversely, the *P. inaequalifolia* essential oil has safrole (32.10%), 11-αH-himachal-4-en-1-β-ol (25.29%), and myristicin (13.29%) as its main chemical constituents [26]. *P. pellucida* essential oil presents as major compounds carotol (26.6–32.0%), dillapiole (25.1–30.2%), and pygmaein (5.5–10.5%) [27]. In another study carried out with the same species, the predominance of β-farnesene (22.2%), β-bisabolene (14.8%), and β-bergamotene (10.7%) in the essential oil was demonstrated [28].

In this context, the present work aims to carry out a seasonal and circadian study analyzing the chemical composition of essential oils and aromas obtained from *P. circinnata* Link var. *circinnata*, and, in addition, to carry out a preliminary toxicity study on *Artemia Salina* and to study in silico the prediction of potential molecular iteration mechanism.

## 2. Results and Discussions

Essential oils’ yields (mg/100 g) obtained from the whole plant devoid of spikes for the seasonal study (July/2010 to May/2011) of fresh and dry samples ranged between 3.5–7.9% and 1.2–2.4%, respectively. Variations in the essential oils’ yields obtained from the fresh plant may have been influenced by climatic variations at the collection time (Table 1). Thus, in July and September were the highest levels, which then decreased in January and March, reaching minimum values. It is also observed that the fresh botanical material showed a yield greater than 50% compared to the dry material.

In a previous study on the essential oil yield from *P. circinnata* Link var. *circinnata* [22], the authors obtained values of 1–2.8%, similar to those observed in this study. In addition, studies have related the maximum and minimum essential oils yields to the rainfall index as one of the factors which added to the period of seasonal collection [29,30,31,32].

The essential oil yields obtained in the months of November and March for the circadian study are shown in Table 2. The botanical material collection was carried out in two periods, evening (M) and afternoon (A). The material was dried by two processes: oven and lyophilization. It is observed that the essential oils’ yields obtained from fresh (F), oven dried (D), and lyophilized (L) materials of collections carried out in the evening and afternoon did not show significant variations owing to the function of time (Table 2). *P. circinnata* Link var. *circinnata* essential oils obtained in the rainy season of march showed yields of 1.2–3.5%. For fresh samples collected at night, the best yield was 3.5%. In the period of years considered to have been without rain, the month of November (dry period) produced fresh samples which had the highest yields, as seen in Table 2, with the highest value being 5.6%. In this study, lyophilization was the technique that most influenced the lowest essential oil yields, with a variation in the yield of 1.2–1.8% for the month of March and 1.6% for November; according to Chua et al. [33], drying methods can influence mass yields and no method is 100% effective for dehydrating plants rich in essential oils. As observed by other authors [34,35,36,37], oven drying and lyophilization can change the samples’ morphological characteristics in relation to being fresh or dried at room temperature, which can hinder the essential oils’ extraction, affecting their mass yield and chemical composition.

### 2.1. Chemical Composition

The relative chemical composition of *P. circinnata* Link var. *circinnata* essential oil whole plant devoid of spikes, fresh, and dry material was different during the seasonal study. The highest concentration of hydrocarbon monoterpenes was obtained from the dry plant, and oxygenated monoterpenes were obtained from fresh samples (Table 3). In total, 38 compounds present in essential oils extracted in the seasonal period were identified, with the majority being myrcene (4.7–16.4%), β-phellandrene (4.3–28.1%), β-elemene (4.3–10%), germacrene D (5.3–13%), and elemicin (1.1–22%). Obtained from collections in July and January (dry and fresh), samples were characterized by the presence of the phenylpropanoid elemicin (13.9–22%), followed by the monoterpene hydrocarbon β-phellandrene (11.1–21.5%), and those obtained by essential oils from dry (September, November, March, and May) and fresh (March and May) botanical materials were characterized by the presence of monoterpene hydrocarbons β-phellandrene (16.5–28.1%) and myrcene (4.7–16.4%), followed by the oxygenated sesquiterpene elemol (4.2–11.2%).

Essential oils from fresh botanical material collected in September and November were characterized by the presence of oxygenated sesquiterpene elemol (15.0–15.1%); followed by sesquiterpene hydrocarbons germacrene D (8.8–11.3%), and β-elemene (8.3–10.0%) (Table 3). The phenylpropanoid elemicin showed a concentration in essential oils of fresh and dry samples from July and January, with 13.9–22.0% variation, slightly decreasing in September (tr-1.1%), and absent in November, March, and May (Table 3).

In Table 4, showing seasonal variation, the chemical components and retention rates obtained from the aromas of the whole plant devoid of spikes of fresh and dry material are listed, as well as the spikes and samples from November, January, March, and May collections. A total of 37 compounds were identified within the months studied. The aroma of dry (March and November) and fresh (November) whole plants was characterized by the presence of myrcene (13.8–20%), β-phellandrene (13.2–19.0%), cubebol (8–10.7%), and elemol (6.2–9.3%); fresh spikes’ aroma (November) was characterized by the presence of myrcene (20.3%), β-phellandrene (11.5%), elemol (12.7%), and *cis*-nerolidol (9.3%); the aroma of the whole plant, both dry (January and May) and fresh (May), was characterized by β-phellandrene (8.6–19.1%), myrcene (8.3–11.8%), δ-cadinene (6.8–15.7%), and elemol (5.0–8.4%); the fresh whole plant aromas (January and March) were characterized by β-phellandrene (16.6–18.7%) and myrcene (13.5–16.9%), in addition to the phenylpropanoid elemicin present in the January collection (12.7%) and the sesquiterpene hydrocarbon dauca-4(11),8-diene (9.2%) present in the March collection; the subgroup was obtained from fresh spikes (January, March, and May) characterized by the presence of methyl eugenol (27.4–31.6%), in addition to the monoterpenes myrcene (12.0–31.7%) and β-phellandrene (7.3–24.4%).

Seasonal variation was observed among the constituents identified in the aromas of the whole plant devoid of spikes in both fresh and dried samples (Table 4), as well as in the whole plant and spikes’ fresh samples obtained from the November, January, March, and May collections. The mono and sesquiterpenes present in the whole plant samples from November showed similar levels in relation to the dry and fresh samples; in January the sesquiterpenes were 50% higher than the monoterpenes in the dry sample, while in the fresh sample the contents were similar, also presenting a higher concentration of phenylpropanoid elemicin (12.7%); in March the sesquiterpenes contents were approximately 15% higher in the dry sample, while in the fresh one the monoterpenes were superior (≈9.0%) to the sesquiterpenes. In May, sesquiterpenes were higher in the fresh samples’ aromas (20%) and predominated in the dry (50%) compared to monoterpenes.

The relationship between the constituents identified in the aromas of the whole plant and the fresh materials’ spikes can be seen in Table 4. The monoterpenes and sesquiterpenes showed similar levels in the months of November, January, and March for the whole plant, the same occurring in the spikes in the months of November and May; the greatest variation in the terpenes class occurred in spikes of March, 7% of sesquiterpenes and 62% of monoterpenes, in the whole plant occurred in May, but there was an inversion, that is, the sesquiterpenes (60%) were superior to the monoterpenes (3%). The presence of the phenylpropanoid class was represented by methyl-eugenol in spikes during the four months of collection (5.4–31.6%) and elemicin in the whole plant in January (12.7%) and in spikes in May (7.0%).

The analysis of essential oil in the circadian study can be seen in (Table 5); in general, 38 compounds were identified in all analyzed fractions, with the predominance of mycrene (4.7–12.1%), β-phellandrene (4.3–28.1%), β-elemene (2.7–10%), germacrene D (5.8–13%), cubebol (0–7.5%) elemol (0–15%), and elemicin with a range of (0–18.3%). *P. circinnata* whole plant devoid of spikes also revealed circadian variation on the chemical composition of fresh and dry samples, mainly in November. In March, there was no significant variation between monoterpenes and sesquiterpenes. The variation was clearly observed in the November collection, in which sesquiterpenes predominated over mono, mainly in the fresh evening sample (82%), ranging from 59–71% in the others. Phenylpropanoid elemicin only appeared in the month of March, in the afternoon collection (2.5 and 4.0%).

In Zoghbi et al.’s [22] study on *P. circinnata* Link var. *circinnata* essential oil, the highest concentrations were myrcene (12.2–31.2%) and β-phellandrene (17.5–25.4%); in Silva et al.’s [23] study, the highest concentrations were myrcene 8.3%, limonene 13.5%, cubebol 9.7%, and elemicin 11.5%. Other *Peperomia* species such as *P. rotundifolia*, *P. pelucida*, and *P. macrostachya* from the Amazon were found to contain major compounds, such as epi-α-bisabolol 15.9%, caryophyllene oxide 12.9%, myristicin 7.6%, aromatic compound 6.6%, and andlimonene 5.4% in *P. macrostachya*, dillapiole 55.3%, (*E*)-caryophyllene 14.3%, and carotol (8.1%) in *P. pellucida*, and decanal (43.3%) in *P. rotundifolia* [38].

#### 2.1.1. Multivariate Analysis

##### Chemical Composition of Seasonal Study

**Figure 1:**
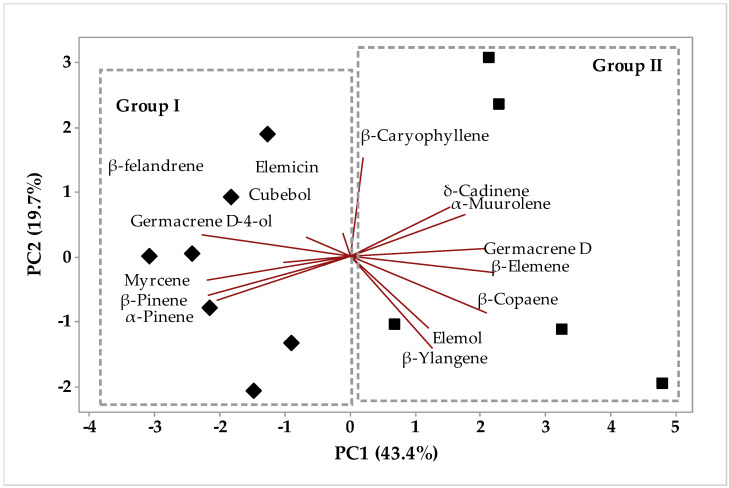
Biplot (PCA) resulting from the analysis of compounds identified in *P. circinnata* Link var. *circinnata* essential oil in the seasonal study.

**Figure 2:**
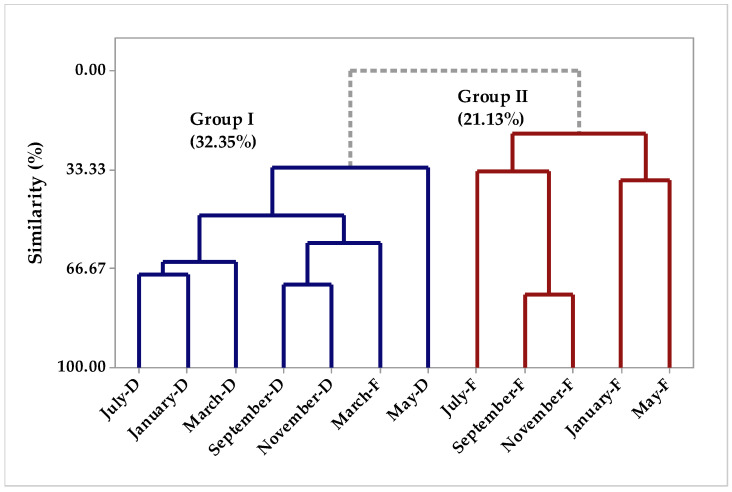
Dendrogram representing the similarity relationship of the compounds identified in *P. circinnata* Link var. *circinnata* essential oil in the seasonal study.

The multivariate analysis PCA (Principal Component Analysis) (Figure 1) and HCA (Hierarchical Cluster Analysis) of the chemical compounds identified in the essential oils different fractions from samples collected between March and November. The first PC1 component explains 43.4% whereas PC2 explains 19.7% of the variations, and the two components add up to 63.1% of variance (Figure 1). The HCA analysis, considering the Euclidean distances and complete bonds (Figure 2), confirmed the formation of two distinct groups. Group 1, with 32.35% similarity, is formed by samples collected from March to November and dried in an oven, plus a sample of fresh *P. circinnata* Link var. *circinnata* collected in March.

Group 2 with 21.13% similarity, shown in Figure 2, was formed by samples collected from May to November without drying treatment (fresh samples). The compounds that characterized group 1 were elemecin, β-phellandrene, germacrene D-4-ol, cubebol, myrcene, β-pineneand, and α-pinene, whereas group 2 was formed by the compounds β-caryophyllene, δ-cadinene, α-muurolene, germacrene D, β-elemene, β-copaene, elemol, and β-ylangene. We also observed that in the present study, seasonality was not the main variable for the groups’ separation, but instead it was the treatment of samples before extraction, which may mean that pre-treatment can be a variable for maintaining the chemical composition without generating losses of compounds by volatilization or degradation.

##### Multivariate Analysis of the Aroma Chemical Composition

**Figure 3:**
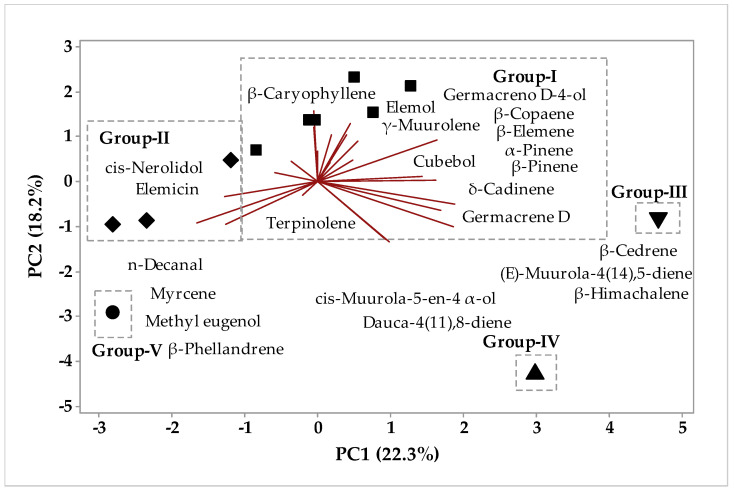
Biplot (PCA) resulting from the analysis of compounds identified in *P. circinnata* Link var. *circinnata* aroma in the seasonal study.

**Figure 4:**
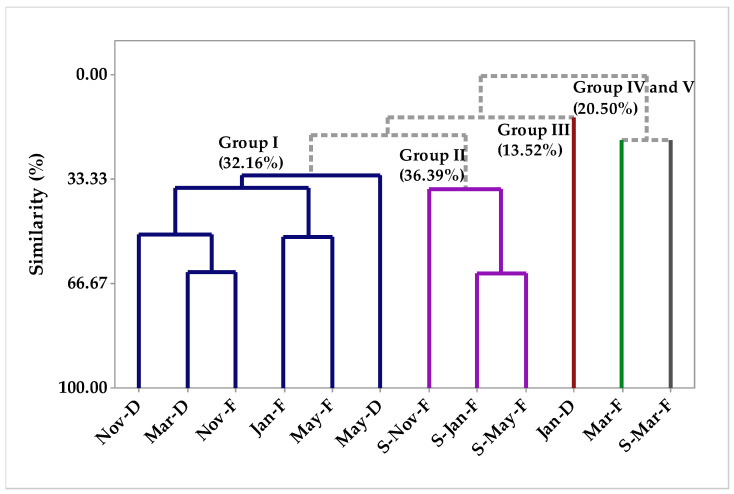
Dendrogram representing the similarity relationship of the compounds identified in *P. circinnata* Link var. *circinnata* aroma in the seasonal study.

Principal component analysis was applied to the aroma of *P. circinnata* Link var. *circinnata’s* whole part and its spikes, and in Figure 3 we observe the principal components analysis, in which the first component explains 23.3% of the variations, whereas the second component explains 18.2%, and the sum of the variances explains 41.1%. Considering the Euclidean distances and complete bonds (Figure 4), in the HCA analysis, the formation of five groups is observed, with group 1 being formed by the samples Nov-D, Mar-D, Nov-F, Jan-F, May-F, and May-D, showing a 32.35% similarity degree, and being characterized by β-caryophyllene, germacrene D, β-elemene, cubebol, γ-muurolene, terpinolene, germacrene D-4ol, β-copaene, δ-cadinene, α-pinene, elemol, and β-pinene compounds. Group 2 (Figure 3), was characterized by the grouping of S-Nov-F, S-Jan-F, and S-May-D samples, showing a similarity of 36.39%, and was characterized by *cis*-nerolidol and elemecin compounds. Group 3 (Figure 3) was formed only by the Jan-D sample, which in the principal component analysis was characterized by β-cedrene, (*E*)-muurola-4(14),5-diene, and β-himachalene. Groups 4 and 5 (Figure 3) were formed by Mar-F and S-Mar-F samples, showing a 20.50% similarity degree for the two samples, being characterized by, *cis*-muurola-5-en-4-α-ol and douca-4(11),8-diene, and β-phellandrene, *n*-decanal, myrcene and methyl eugenol compounds, respectively.

##### Multivariate Analysis of the Circadian Study Chemical Composition

**Figure 5:**
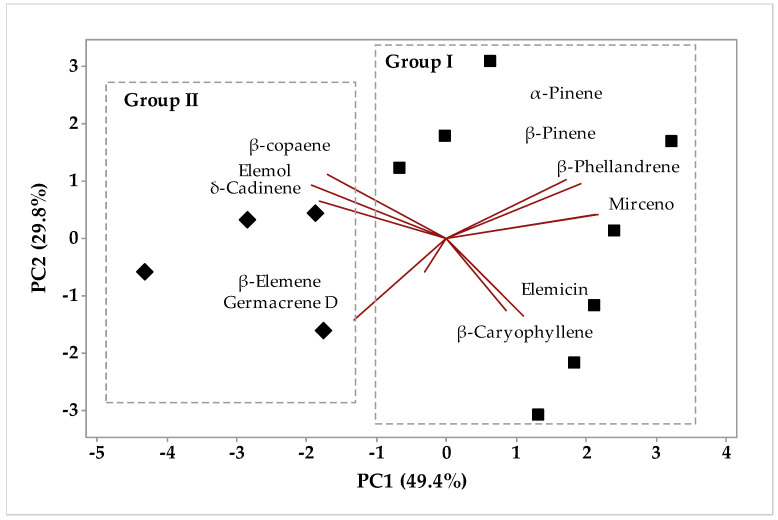
Biplot (PCA) resulting from the analysis of compounds identified in *P. circinnata* Link var. *circinnata* essential oil in the circadian study.

**Figure 6:**
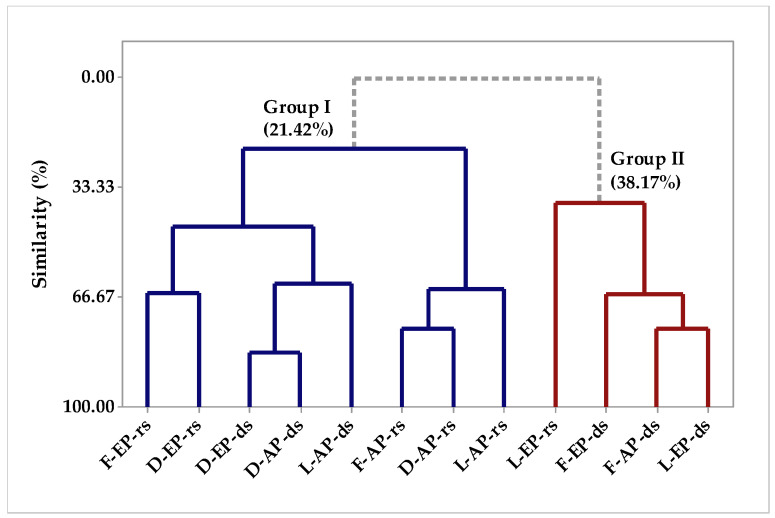
Dendrogram representing the similarity relationship of the compounds identified in *P. circinnata* Link var. *circinnata* oil in the circadian study (fresh (F), dried (D), lyophilized (L), evening period (EP), afternoon period (AP), rainy season (rs), and dry season (ds)).

In the circadian study, the essential oils of *P. circinnata* Link var. *circinnata* collected in the evening and afternoon, in the winter and summer, when applying principal component analysis (PCA), it is observed that PC1 explained 49.4% and PC2 explained 29.8% of the analyzed variables, while the sum of the variances PC1 and PC2 added up to 79.2% (Figure 5). Figure 6 brings the Hierarchical Cluster Analysis (HCA); the formation of two groups can be observed, group 1 being formed by the grouping of samples collected in the evening and afternoon, fresh and dried in greenhouses as F-EP-rs, D-EP-rs, D-EP-ds, D-AP-ds, L-AP-ds, F-AP-rs, D-AP-rs, and L-AP-rs. In Figure 5, we can see that group 1 was characterized by the compounds that had the highest weights for analysis, such as β-caryophyllene, elemecin, mircene, β-phellandrene, β-pineneand, and α-pinene. Group 2 (Figure 6) was formed by agglutination of L-EP-rs, F-EP-ds, F-AP-ds, and L-EP-ds samples. In Figure 5, using PCA, group 1 was characterized by the presence of the components β-copaene, elemol, δ-cadinene, β-elemene, and germacrene D, and group 2 was characterized by α-pinene, β-pinene, β-phellandrene, mircene, elemecin, and β-caryophyllene. In Pirbalouti’s [40] work, the increase in drying temperature decreased the concentrations of α-pinene, sabinene, β-myrcene, and β-phellandrene in the basil essential oil.

### 2.2. Cytotoxicity Bioassay in Artemia Salina

In the control group tests there was no mortality; therefore, the use of DMSO is viable as a solvent for the assay. LC_50_ values were calculated by converting the percentage of larvae mortality into probits, thus making it possible to trace the equation as a function of concentration values on a logarithmic scale (Table 6). Furthermore, studies have shown that there is a strong correlation between in vitro toxicity results using *A. salina* and in vivo study using natural products [41,42].

The tested oils showed a mortality rate ranging from 100 to 10%, according to the concentration range. These concentrations ranged from 50 to 5 μg·mL^−1^ for samples from July (fresh and dry) and January (fresh), from 100 to 10 μg·mL^−1^ for samples from September, November, and January (dry material), and March and May (fresh and dry) (Table 6). The sample with the lowest toxicity was from the month of May (fresh) which had LC_50_ (51.55 ± 2.12 μg·mL^−1^) (Table 6), which presented sesquiterpenic hydrocarbons as its major constituents; the results of the present work were close to those obtained for *Schinusmolle* L. essential oil rich in α-phellandrene, β-phellandrene, β-myrcene, limonene, and α-pinene, where it presented LC_50_ 47 and 67 μg·mL^−1^ for leaf and fruit. In the present work, the sample that had the highest toxicity was from July (dry), that had LC_50_ (14.45 ± 0.25 μg·mL^−1^) (Table 6), which presented as main constituents β-phellalandrene and elemicin (Table 3). In general, all the essential oil fractions tested had LC50 < 100 μg·mL^−1^ values, which means that essential oils have high toxicity [43], and values > 1000 μg·mL^−1^ can be considered of low toxicity [44].

Toxicity tests in *A. Salina* performed using *Hyptis suaveolens* (L.) Poiteau (Lamiaceae) essential oil [45] presented LC_50_ values similar to those obtained in the present work, whereas the *Garcinia mangostana* essential oil obtained LC_50_ of 1.70 µg·mL^−1^ and 5.15 μg·mL^−1^ for leaves and stem [46], and *Ferulago trifida* had LC_50_ 1.1 ± 0.3 μg·mL^−1^ [47]; these toxicities were higher than those presented by *P. circinnata* Link var. *circinnata* essential oils. Essential oils rich in phenylpropanoids such as cloves, demonstrated LC_50_ value of 0.5993 ± 0.0464 μg·mL^–1^ [48], which is higher than the tests of the major substances tested separately; this may be related to the synergistic effect of the substances present in the essential oil [49,50,51]. According to Radulović et al. [52], essential oils that have high toxicity values must be carefully managed to avoid intoxication. In a study carried out with three species of *Peperomia*, the authors obtained LC_50_ results of 1.9 ± 0.1 μg·mL^−1^ for *P. rotundifolia* essential oil, 2.4 ± 0.5 μg·mL^−1^ for *P. pellucida* extract, and 9.0 ± 0.4 μg·mL^−1^ for *P. macrostachya* essential oils.

### 2.3. In Silico Evaluation of Interaction with AChE

**Figure 7:**
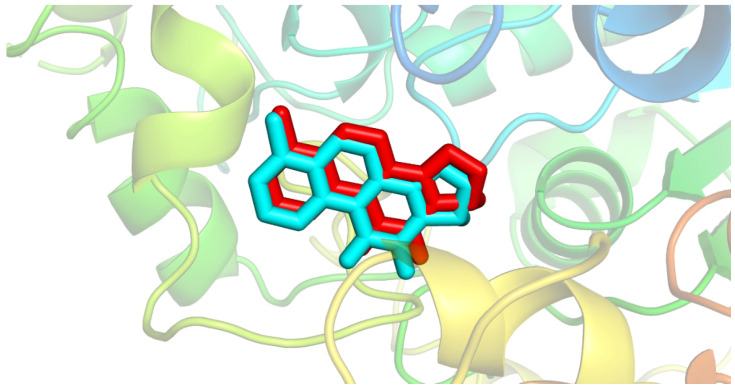
Structure obtained by redocking (blue) overlaying the crystallographic binder (red).

Previous studies have successfully reported the use of in silico approaches to evaluate the interaction of naturally occurring compounds that have molecular targets of pharmacological and toxicological interest [53,54,55,56]. For this reason, molecular docking was used to investigate the interactions established between β-elemene and elemicin with AChE. This enzyme has been reported as a target for *A. salina* [21,57]. The binding mode and interactions established in the complex can be seen in Figure 7.

Before performing the docking of the molecules of interest, it was necessary to validate the protocol described in the methodology. To develop the methodology, we first tried to reproduce the binding mode of the crystallographic ligand performing its redocking. For this, the ligand of the PDB 4M0E was deleted [58] and then it was redocked. To assess the binding mode’s reproducibility, the ligand interacting conformation was evaluated by comparing the redocked structure with the crystal structure. The evaluation of the obtained complexes was carried out using the root-mean-square deviation RMSD between the ligands. According to the literature, for the docking protocol to be validated, the RMSD between the redocked and the crystallographic ligand must be less than 2 Å [59,60,61,62]. In our results, an RMSD of 1.42 Å was obtained. In Figure 7, it is possible to visualize the compounds overlapping. After protocol validation, the docking between β-elemene and elemicin was performed.

The MolDock Score obtained was −91.61 Kcal/mol for the complex formed by β-elemene and −90.19 Kcal/mol for the system established with elemicin. In these binding poses the ligands were able to form an interaction with residues from the enzyme catalytic cavity. These interactions are able to favor the compounds inhibitory capacity.

**Figure 8:**
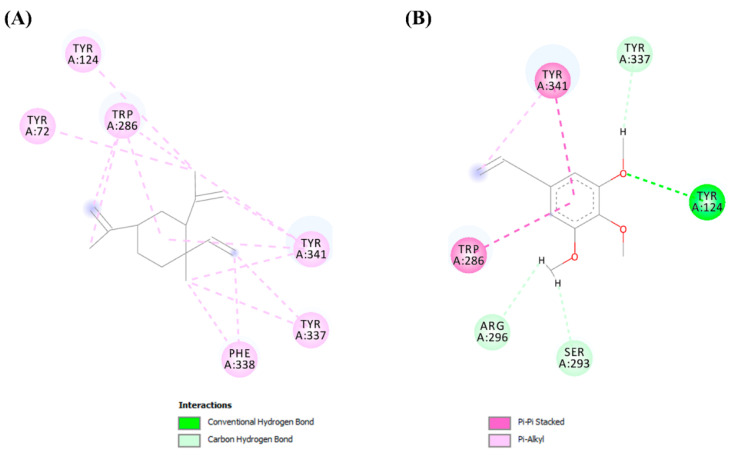
Interactions established in the complexes established with (**A**) β-elemene and (**B**) elemicin.

The AChE binding cavity is formed by three subsites, the anionic subsite (Trp86, Tyr133, Tyr337, and Phe338), acyl pocket (Phe295 and Phe297), and oxyanion hole (Gly121, Gly122, and Ala204) [63,64]. In Figure 8A,B, it shows that the two ligands are able to interact with residues present in the anionic subsite. With residues from this subsite, the β-elemene compound interacted with Tyr337 and Phe338 through hydrophobic interactions of the pi-alkyl type. The elemicin ligand interacted with Tyr337 through hydrogen bonds. Besides these, other interactions were established with the ligands in the binding pocket. The β-elemene also interacted with Tyr124, Tyr72, Trp286, and Tyr341. All these interactions were hydrophobic of the pi-alkyl type. Elemicin formed pi-pi type hydrophobic interactions with Tyr341 and Trp286, as well as hydrogen bonds with Tyr124, Figure 8A,B.

## 3. Materials and Methods

### 3.1. Collection of Botanical Material for Seasonal and Circadian Study

*P. circinnata* Link var. *circinnata* collections occurred bimonthly, from July to May, in the evening, always on the 15th day at 8 am in Belém, Pará, and the samples were removed from the mango tree trunks near the Emílio Goeldi Zoo and Botanical Park. In November and March, collections were carried out in the evening and afternoon (8 am and 5 pm) for the circadian study. This specimen was identified by comparison with an authentic voucher (MG 172736) deposited in the Emılio Goeldi Museum herbarium, city of Belém, Pará state, Brazil.

### 3.2. Processing of Botanical Material

Whole plants devoid of spikes were divided into two parts; the fresh material was cut into small parts and subjected to the hydrodistillation process. The remainder was dried in an air circulation oven at room temperature for five days, then ground, homogenized, weighed, and subjected to hydrodistillation. Drying for the circadian study was achieved by two processes: oven (ventilation) and lyophilization.

#### 3.2.1. Extraction Methods

##### Hydrodistillation

For the essential oil extraction process in circadian and seasonal studies, 40 g of fresh and dry sample from *P. circinnata* Link var. *circinnata* were dried in an air circulation oven and then subjected to hydrodistillation. The same proportion of water in relation to plant material was used, according to the methodology described by [53,65].

##### Simultaneous Distillation–Extraction

For aroma extraction, 10 g of sample from *P. circinnata* Link var. *circinnata* was used, then mixed with water (20 mL) and subjected to simultaneous distillation–extraction (SDE) for 3 h, using a Chrompack Micro-Steam Distillation Extractor (Likens-Nickerson) and pentane (2 mL) as organic mobile phase, as described by [66].

### 3.3. Identification of Chemical Constituents

The chemical composition of *P. circinnata* Link var. *circinnata* essential oils and aromas was analyzed by Gas Chromatography coupled with Mass Spectrometry, using a Thermo DSQ-II system equipped with a DB-5MS silica capillary column (30 m × 0.25 mm; 0.25 μm) with temperature program: 60–240 °C, using gradient of 3 °C/min; injector temperature: 240 °C; carrier gas: helium (linear velocity of 32 cm/s, measured at 100 °C); splitless injection flow (0.1 mL of a 2:1000 sol. of *n*-hexane); temperature of ion source and other parts at 200 °C. The quadrupole filter swept in the range of 39 to 500 daltons every second. Ionization was achieved by the electronic impact technique at 70 eV. Volatile components identification was based on the linear retention index (Kováts Index) calculated in relation to the retention times of a homologous series of *n*-alkanes (C_8_–C_40_) according Van den Dool and Kratz [67] and on the fragmentation pattern observed in the mass spectra, by comparison in the data system and literature libraries [39,68]. Quantitative data regarding the volatile constituents were obtained by peak-area normalization using a FOCUS GC/FID, as previously reported by our research group [69].

### 3.4. Preliminary Toxicity Bioassay with Artemia Salina Leach

For the toxicity tests, the sample with the highest mass yield was selected. The preliminary toxicity bioassay test of *P. circinnata* Link var. *circinnata* essential oil in *A. salina* Leach was performed as described in the literature [57,70,71]. The essential oil was prepared at concentrations of 100, 50, 20, 10, 5, and 1 μg·mL^−1^ were used, from fresh and dry samples obtained in the seasonal study in the months of July, September, November, January, March, and May. A total of ten *Artemia salina* larvae were added to each test flask with the aid of automatic micropipettes. Brine water (artificial) and DMSO were used as solvents with a 95:5 ratio. In the control group and the positive group with lapachol, the same solvent was used for the samples and larvae under the same conditions as the bioassay. After 24 h of contact between the larvae and the sample solution, the dead larvae were counted (in each concentration), and the mortality rate and the IC_50_ value were calculated using the Probitos statistical method. All the experiments were performed in triplicate (*n* = 3).

### 3.5. Molecular Docking

For molecular docking studies, the compounds β-elemene (PubChem CID 9859094) and elemicin (PubChem CID 10248) were obtained from Pub Chem database (https://pubchem.ncbi.nlm.nih.gov/, accessed on 20 May 2021). Then, their structures were optimized with B3LYP/6-31G* [72] using Gaussian 09 software [73].

We used the molecular method to evaluate the compounds interaction mode with Acetylcholinesterase (AChE). For this we used the Molegro Virtual Docker (MVD) 5.5 software [74], and the crystal structure used as a molecular target can be found in the Protein Data Bank (http://www.rcsb.org, accessed on 20 May 2021) using the ID: 4M0E [58].

The MolDock Score (GRID) scoring function was used with Grid resolution of 0.30Å and 5Å radius encompassing the entire connection cavity. The MolDock SE algorithm was used with number of runs equal to 10, 1500 max interactions, and max population size equal to 50. The maximum evaluation of 300 steps with neighbor distance factor equal to 1 and energy threshold equal to 100 was used during the molecular docking simulation.

### 3.6. Statistical Analysis

Multivariate analysis was performed according to the methodology described by [69,75], where Minitab 17^®^ software (free version, Minitab Inc., State College, PA, USA) was used. The variables were the essential oils’ chemical constituents. The raw data were first standardized to have the same “weight”. The Principal Component Analysis (PCA) was obtained using the software configuration “correlation” of the matrix type. In the Hierarchical Cluster Analysis (HCA) of the samples, the Euclidean distance options were used for distance measurement and complete connection for connection method.

## 4. Conclusions

The highest essential oil yield from the seasonal analysis of *P. circinnata* Link var. *circinnata* during the study period was found in the fresh plant, especially in July, that is, in the dry season, which may mean that low humidity contributes to oil production. Multivariate analysis allowed to observe the similarities and differences between the chemical compositions in the different periods studied, showing that, according to the period and time of collection, there is a qualitative and quantitative change in the chemical composition. In the circadian study of the months of November and March, there were no significant variations in oil yield in the evening and afternoon collections. The essential oils obtained from fresh and dry plants showed a quantitative difference, in relation to the more volatile constituents (monoterpenes). Seasonal variation can be seen in relation to the presence of the phenylpropanoid elemicin in the months of July and January; as well as the oxygenated sesquiterpene elemol. These qualitative and quantitative variations of the oil are affected by seasonality and, consequently, are reflected in the biological activities evaluated, as at all concentrations we can observe that there was a low LC50 representing a potential biological activity of the samples. In the in silico study, it was observed that in the complex formed between AChE-β-elemene and AChE-elemicin, the interactions formed had residues present in the anionic subsite of the enzyme, in addition to interactions with other residues of the enzyme. Most of these interactions were hydrophobic and helped to maintain the systems formed by molecular interactions.

## Figures and Tables

**Table 1:** *P. circinnata* var. *circinnata* essential oil (EOs) yield from seasonal study in (%).

	July		September		November		January		March		May	
	D	F	D	F	D	F	D	F	D	F	D	F
% EOs	2.40	7.90	2.00	4.30	2.40	5.60	1.70	3.60	1.50	3.50	1.20	4.20

F = fresh, D = dry.

**Table 2:** *P. circinnata* Link var. *circinnata* essential oil yield from circadian study.

	March						November					
	Evening			Afternoon			Evening			Afternoon		
	F	D	L	F	D	L	F	D	L	F	D	L
% oil	3.5	1.5	1.8	2.9	1.5	1.2	5.6	2.5	1.6	5.1	2.3	1.6
Moisture	92.4	22.0	43.5	90.9	30.6	49.2	91.1	37.4	45.0	88.9	12.7	33.6

Evening, afternoon, fresh (F), oven drying (D), and lyophilization (L).

**Table 3:** Seasonal variation on the chemical composition of *P. circinnata* Link var. *circinnata* essential oils in the months of July, September, November, January, March, and May, fresh (F) and oven dried (D). The concentration values of the compounds are (%).

Constituents	RI_L_	RI_C_	Jul-D	Jul-F	Set-D	Set-F	Nov-D	Nov-F	Jan-D	Jan-F	Mar-D	Mar-F	May-D	May-F
α-pinene	932	932	2.9	1.4	3	0.5	2.5	0.8	1.7	1.1	2.4	3.5	0.7	0.2
β-pinene	974	978	3.2	2.2	3.3	0.8	2.9	1.2	2.3	1.6	3	3.2	1.4	1
mycrene	988	987	10.8	7	15.3	6.2	10.9	5.5	12.1	7.9	13.9	16.4	9.2	4.7
ρ-mentha-1 (7),8-diene	1003	1006	0.3	0.2	0.6		0.8	0.3	0.6	0.5	0.7	1.1	1.2	0.9
β-phellandrene	1025	1029	21.5	14	16.5	4.3	20.6	8	16.1	11.1	23.3	28.1	24.9	16.5
terpinolene	1086	1083	1.2	1	1.4	1	1.8	1	1.4	1.4	1.1	2.5	2.1	1
*n*-decanal	1201	1205	1.2	1.8	1.7	2	0.7		1.4	2.2	0.9		1.7	0.7
α-ylangene	1373	1366	0.4	1.9	0.3	0.4	0.2	0.4	0.2	0.2	0.5	0.7	0.5	0.8
α-copaene	1374	1373	1.6		1.5	2.8	1.3	2.3	1.5	1.9	1.6	2	1.2	2.7
β-elemene	1389	1387	5.9	7.3	6	10	4.3	8.3	5.1	6.4	5.9	2.7	5.9	7.7
methyl eugenol	1403	1401							0.2	0.3	0.3	0.4	0.1	
dodecanal	1408	1409	0.7	1.2	0.7	1.3	0.4	0.9	0.7	1.3	0.5	0.6	0.6	0.7
β-ylangene	1419	1417	1.5	2	1.5	3	1.8	2						
β-caryophyllene	1417	1418	1	0.6	1	1	1	1.6	2.3	3.1	2.2	2.2	2.5	4.1
β-cedrene	1419	1420	0.6	0.4	0.3		0.2	0.4	0.5	0.3	0.5	0.8	0.5	1.1
β-copaene	1430	1426	1.8	2.6	2.3	3.7	2.3	3.2	1.7	2.2	1.6	2.1	1.4	2.3
α-neo-clovene	1452	1447	0.6		0.7	1.1	1	1	0.5	1.3	0.6	0.6	0.5	1.1
α-humulene	1452	1451	0.6	0.4	0.5	0.8	0.6	0.9	0.5	0.7	0.5	0.3	0.6	1
Alloaromadendrene	1458	1455	0.4	0.8	0.5	0.8	0.5	0.7	0.5	0.5	1.1	0.5	0.4	0.8
*E*-β-farnesene	1454	1464	0.6	0.3	0.2	0.1	0.4	0.4	0.4	0.4		0.3	0.4	1
γ-muurolene	1478	1471	1	1.1	1.4	1.5	0.8	1.6	1.3	0.8	1.4	1.5	1.4	2.4
germacrene D	1484	1478	5.8	7.3	6.8	11.3	6.8	8.8	5.8	7.8	6.6	6.2	5.3	13
*trans*-muurola-4 (14),5-diene	1493	1487	0.4	0.5	0.6	0.9	0.7	1.5	0.6		0.5	0.6	0.5	0.9
epi-cubebol	1493	1492	1	1	1.2	1.2	1.1	1.2	1.4		1.8	0.8	1.5	1.8
α-muurolene	1500	1495	1	1.3	1.2	2.5	1.4	2.2	1.1	4.1	1.2	1.4	1.1	1.9
cubebol	1514	1512	5.1	0.4	4.6	3.7	3.7	4	6.4		7.5	2.8	5.6	4.9
δ-cadinene	1522	1515	2.3	1.8	2.3	5.7	5.2	5.5	2.1	9.4	2.1	3.6	5	4.6
zonarene	1528	1518										0.4	1.3	0.3
*cis*-nerolidol	1531	1521	0.6	0.3			1.7	0.8						
elemol	1548	1542	0.1	2.9	10	15.1	11.2	15	0.5		6	4.6	4.4	4.2
elemicin	1555	1547	13.9	22	1.1				18.3	18.1				
germacrene D-4-ol	1574	1573	0.6	0.7	0.4	0.2	1.4	0.2	0.4	0.1	0.6		3.9	
junenol	1618	1603	0.7	0.7	0.7	0.9	0.6	1.2	0.8	1	0.9	0.6	0.5	1.1
1.10-di-epi-cubenol	1618	1623	0.4	0.4	0.7	1.3	0.7	1.6	0.8	1.4	0.7	0.8	0.5	1.1
epi-α-cadinol	1638	1633			0.1		0.6		0.9	0.9			0.4	0.6
epi-α-muurolol	1640	1639	0.6	0.5	0.8	1.8	0.6	1.9	0.7	0.7		1	0.8	0.7
α-muurolol	1644	1642	0.4	0.6	0.7	1	0.7	1	0.2	0.8		0.5	0.5	
α-cadinol	1652	1651	0.8	0.7	1.2	1.5	2	1.9	1.5	2	0.8	0.4	1.9	1
Monoterpene hydrocarbons			39.9	25.8	40.1	12.8	39.5	16.8	34.2	23.6	44.4	54.8	39.5	24.3
Oxygenated monoterpenes			1.2	1.8	1.7	2	0.7		1.4	2.2	0.9		1.7	0.7
Sesquiterpenes Hydrocarbons			26.1	28.6	27.1	45.6	30.2	41.6	24.1	39.1	26.3	25.9	28.5	45.7
Oxygenated sesquiterpenes			9.7	7.9	20.4	26.7	22.6	28	13.6	6.9	18.3	11.5	20	15.4
Phenylpropanoids			13.9	22	1.1				18.5	18.4	0.3	0.4	0.1	
Others			0.7	1.2	0.7	1.3	0.4	0.9	0.7	1.3	0.5	0.6	0.6	0.7
Totals			91.5	87.3	91.1	88.4	93.4	87.3	92.5	91.5	90.7	93.2	90.4	86.8

RIC: retention index (on DB-5MS column); RIL: literature retention index (Adams [39]).

**Table 4:** Seasonal variation on the chemical constituents obtained from the samples’ (fresh and dry) aroma of *Peperomia circinnata* collected in the months of November, January, March, and May (fresh (F), oven dried (OD), and fresh spike (S)). The concentration values of the compounds are (%).

			Whole Dry Plant				Whole Fresh Plant				Fresh Spike			
Constituents	RI_L_	RI_C_	Nov-D	Jan-D	Mar-D	May-D	Nov-F	Jan-F	Mar-F	May-F	Nov-F	S-Jan-F	S-Mar-F	S-May-F
α-pinene	932	932	5.2	3.9	5.2	1.6	4.8	3	5.2	1.3	3.3	1.4	1.6	1.3
β-pinene	974	978	4.3	3	4.3	1.8		2.1	4.1	1.9	0.7	0.5	1	0.4
mycrene	988	987	15.4	9.5	13.8	11.8	20	13.5	16.9	8.3	20.3	22.9	31.7	12
ρ-mentha-1 (7),8-diene	1003	1006	1.3	1.2	2.1	1.6	1.3	0.7	1.4	1.5	0.4	0.6	1.1	
β-felandrene	1025	1029	17.2	8.6	13.2	19.1	19	16.6	18.7	14	11.5	7.3	24.4	11.2
terpinolene	1086	1083	2.4	2	2.1	3.1	1.9	1.9	2.6	2	4.2	2.1	0.8	2.6
Octen-3-yl acetate	1110	1106	0.6	1.1	0.8	1.4	0.3	0.3		0.8	0.8	0.5	1.1	0.6
*n*-decanal	1201	1205	0.7	2	1.7	2.3	0.7	2.4	1.1	1	4.4	6.5	1.6	4.5
α-copaene	1374	1373	0.8	1.5	1.5	0.7	1.4	1.1	1.4	1.8	0.4	0.6	0.1	0.3
β-bourbonene	1387	1379	0.3	1	0.4	0.3	0.4	0.4	0.3	0.6	0.3	0.5	0.1	0.2
β-elemene	1389	1387	2.9	5.6	4.9	4.1	3.6	4.5	2.5	5.9	3.6	7.6	1.3	4.4
methyl eugenol	1403	1399				0.1	0.3	0.1		0.2	5.4	28.8	27.4	31.6
dodecanal	1408	1409	0.4	0.9	0.8	0.4	0.5	1.2	0.1	0.7	1.2	1.8	0.2	0.9
β-caryophyllene	1417	1415	2		2.5	1.6	2.1	2.3	0.2	3.6		1.1	0.2	0.7
β-cedrene	1419	1419	0.2	5.2	0.5	0.3	0.4	0.4	1.9	0.8				
β-copaene	1430	1426	2.2	3.6	1.3	0.8	1.8	1.2	0.7	2.4	1.4	0.6	0.1	0.6
α-neo-clovene	1452	1447	0.8			0.6	0.8	1	0.3	1.5	0.2	0.1		
α-humulene	1452	1450	0.5	1.4	1	0.4	0.4	0.5		0.8	0.2	0.1		0.1
γ-muurolene	1478	1474	5			1	1.3	0.6	0.4	1.9		0.3	0.1	0.2
*trans*-4,10-epoxy-amorphane	1478	1473	0.5		1.8							0.1	0.1	0.1
germacrene D	1484	1481	0.4		4.9	3.5	5.7	5	1.7	8.6	2	1.5	0.2	1.2
*trans*-muurola-4 (14),5-diene	1493	1489		6.5	0.4	0.4	0.6	0.6	5.4	0.9		0.2	0.1	0.3
epi-cubebol	1493	1494	1	0.6	2.5	1	1.6		0.4	1.8	0.5	0.7	0.3	
α-muurolene	1500	1496	0.1		0.8	0.7	1	1.5		1.5	0.5	0.4	0.1	0.4
*trans*-β-guaiene	1502	1501			0.2				1.6	0.3				
β-himachalene	1500	1500		3.4				0.1	1			0.1		0.1
cubebol	1514	1518	8	0.9	10.6		10.7			2.9		2.6	1.4	1.3
δ-cadinene	1522	1515		8		15.7			0.3	6.8	2.8	0.8	0.6	3.4
zonarene	1528	1518							0.2	0.4			0.1	1.5
*cis*-nerolidol	1531	1521	3					5.4			9.3	0.1		
dauca-4 (11),8-diene	1530	1524			0.2			0.2	9.2	0.7		0.1		0.2
elemol	1548	1547	9.3	8.4	6.2	5.4	7.6	3.2	0.2	5	12.7	3.7	0.3	4.5
elemicin	1555	1555						12.7		2.4				7
*cis*-muurol-5-en-4 α-ol	1559	1572		0.4					7.7	0.7		0.2	0.1	0.2
germacrene D-4-ol	1574	1575	3	2.5	2.2	7.8	0.7	0.9		0.6	1.4	0.1		0.5
junenol	1618	1603	0.4	1.4	1.4	0.6	0.9	0.7	0.2	1.1		0.1	0.2	0.2
α-cadinol	1652	1651	1.2	1	0.5	1	1	1.5	0.6	1.8	2.9	1.3		1.6
Monoterpene hydrocarbons			45.8	28.2	40.7	39	47	37.8	48.9	29	40.4	34.8	60.6	27.5
Oxygenated monoterpenes			1.3	3.1	2.5	3.7	1	2.7	1.1	1.8	5.2	7	2.7	5.1
Sesquiterpenes Hydrocarbons			15.2	36.2	18.6	30.1	19.5	19.4	27.1	38.5	11.4	14	3	13.6
Oxygenated sesquiterpenes			26.4	15.2	25.2	15.8	22.5	11.7	9.1	13.9	26.8	8.9	2.4	8.4
Phenylpropanoids						0.1	0.3	12.8	0	2.6	5.4	28.8	27.4	38.6
Others			0.4	0.9	0.8	0.4	0.5	1.2	0.1	0.7	1.2	1.8	0.2	0.9
Totals			89.1	83.6	87.8	89.1	90.8	85.6	86.3	86.5	90.4	95.3	96.3	94.1

RIC: retention index (on DB-5MS column); RIL: literature retention index (Adams [39]).

**Table 5:** Chemical constituents obtained from the circadian study of *P. circinnata* Link var. *circinnata* (fresh (F), oven dried (D), lyophilized (L), evening period (EP), afternoon period (AP), rainy season (rs), and dry season (ds)). The concentration values of the compounds are (%).

Constituents	RI_L_	RI_C_	FEP-rs	FAP-rs	D-EP-rs	D-AP-rs	L-EP-rs	L-AP-rs	FEP-ds	FAP-ds	D-EP-ds	D-AP-ds	L-EP-ds	L-AP-ds
α-pinene	932	932	3.5	1.8	2.4	2.4	1.1	1.8	0.8	1.1	2.5	2.1	1.7	2.7
β-pinene	974	978	3.2	2.1	3	2.7	1.3	2.6	1.2	1.9	2.9	2.5	2.2	3.7
myrcene	988	987	16.4	13.8	13.9	11.4	6.7	9.4	5.5	6.3	10.9	9.8	9	11.3
ρ-mentha-1 (7),8-diene	1003	1006	1.1	1	0.7	1	0.5	0.8	0.3	0.61	0.8	0.8	0.6	1.2
β-phellandrene	1025	1029	28.1	24.6	23.3	23.8	15.6	18.3	8	15.6	20.6	18	12.6	24
terpinolene	1086	1083	2.5	1.1	1.1	1.6	1.2	1.5	1	1.4	1.8	1.5	1.2	1.7
*n*-decanal	1201	1205	1.3	1.3	0.9	0.9	1.1	1.7		0.6	0.7	0.6	0.8	0.6
α-copaene	1374	1373	2	2	1.6	1.7	2.3	2	2.3	1.7	1.3	1.6	2	1.2
β-elemene	1389	1387	2.7	6.3	5.9	6		7.1	8.3	5.2	4.3	5	6.3	3.2
methyl eugenol	1403	1399	0.4	0.1	0.3	0.4	0.2							
dodecanal	1408	1409	0.6	0.7	0.5	0.5	0.7	1	0.9	0.4	0.4	0.3	0.5	0.4
β-caryophyllene	1417	1415	2.2	2.9	2.2	2.5	3.2	2.9	1	1.2	0.8	0.8	1	0.8
β-ylangene	1419	1419							2.6	2	2	2	2.3	1.8
β-copaene	1430	1426	2.1	1.2	1.6	1.5	2.3	1.4	3.2	2.8	2.3	2.4	2.7	3.2
α-neo-clovene	1452	1447	0.6	1.1	0.6	0.6	0.9	0.8	1	0.9	1	1.1	0.9	1
alloaromadendrene	1458	1456	0.5	0.8	1.1	0.6	0.7	1.2	0.7	0.6	0.5	0.5	0.6	1
γ-muurolene	1478	1474	1.5	2	1.4	1.4	2	1.5	1.6	1.1	0.8	0.9	1.2	0.8
germacrene D	1484	1481	6.2	8.4	6.6	7.3	9.1	8.4	8.8	8	6.8	7	7.9	5.9
(*E*)-muurola-4 (14),5-diene	1493	1489	0.6	0.6	0.5	0.7	0.7	0.7	1.5	0.9	0.7	0.7	0.9	0.4
epi-Cubebol	1493	1494	0.8	1.5	1.8	1.8	2.2	2	1.2	1.1	1.1	1.4	1.4	1.1
α-muurolene	1500	1496	1.4	1.6	1.2	1.5	2	1.8	2.3	1.7	1.4	1.5	1.9	1.2
δ-cadinene	1522	1515	3.6	3.5	2.1	3.3	4.6	3.6	5.5	6.2	5.2	4.3	4.7	5.4
cubebol	1514	1518	2.8	4.5	7.5	6.5	6.5	6.7	4	3.5	3.7	5.4	3.9	1.8
*cis*-nerolidol	1531	1521					0.4		0.8	1.5	1.7	1	0.4	1.2
elemol	1548	1548	4.6	1	6	2.4	8.8	0.5	15	13.2	11.2	13.3	12.7	8.8
elemicin	1555	1555		2.5		4		8						
germacrene D-4-ol	1574	1575			0.6	0.4	0.4	0.4	0.3	1	1.4	0.9	0.3	0.9
junenol	1618	1603	0.6	1	0.9	1.1	1.5	1.4	1.2		0.6	0.7	1	0.6
1.10-di-epi-Cubenol	1618	1623	0.8	0.9	0.7	0.9	1.4	1	1.6	1.2	0.7	0.9	1.5	0.9
epi-α-Cadinol	1638	1633			0.4	0.7	1	0.7		1.9	0.6			0.8
epi-α-muurolol	1640	1639	1	0.6	0.4	0.3	0.7	0.6	1.9	0.9	0.6	1.3	1.9	1
α-Muurolol	1644	1642	0.5	0.5	0.6	0.5	1	0.6	1		0.7	0.9	1.2	0.9
α-Cadinol	1652	1651	0.4	1	0.8	0.5	2	1	1.9	2.9	2	1.7	2	3.4
Monoterpene hydrocarbons			54.8	44.4	44.4	42.9	26.4	34.4	16.8	26.91	39.5	34.7	27.3	44.6
Oxygenated monoterpenes			1.3	1.3	0.9	0.9	1.1	1.7		0.6	0.7	0.6	0.8	0.6
Sesquiterpenes Hydrocarbons			23.4	30.4	24.8	27.1	27.8	31.4	38.8	32.3	27.1	27.8	32.4	25.9
Oxygenated sesquiterpenes			11.5	11	19.7	15.1	25.9	14.9	28.9	27.2	24.3	27.5	26.3	21.4
Phenylpropanoids			0.4	2.6	0.3	4.4	0.2	8						
Others			0.6	0.7	0.5	0.5	0.7	1	0.9	0.4	0.4	0.3	0.5	0.4
Totals			92	90.4	90.6	90.9	82.1	91.4	85.4	87.41	92	90.9	87.3	92.9

RIC: retention index (on DB-5MS column); RIL: literature retention index (Adams [36]).

**Table 6:** Preliminary toxicity of *P. circinnata* Link var. *circinnata* essential oils with *A. salina*.

Essential Oil	Concentration (μg·mL^−1^)	Mortality (%)	LC_50_ (μg·mL^−1^)
	50	100	
JulF	25	80	17.66 ± 0.33
	10	10	
	5	0	
	50	100	
JulD	25	100	14.45 ± 0.25
	10	20	
	5	0	
	100	100	
SetD	50	80	35.11 ± 0.93
	25	40	
	10	0	
	100	100	
NovD	50	100	26.32 ± 0.00
	25	90	
	10	0	
	50	100	
JanF	25	70	18.29 ± 0.00
	10	10	
	5	0	
	100	100	
JanD	50	100	23.37 ± 2.55
	25	100	
	10	0	
	100	100	
MarF	50	100	26.87 ± 0.47
	25	80	
	10	0	
	100	100	
MarD	50	100	21.90 ± 0.00
	25	100	
	10	0	
	100	80	
MayF	50	50	51.55 ± 2.12
	25	40	
	10	0	
	100	100	
MayD	50	80	33.07 ± 3.80
	25	50	
	10	0	

## Data Availability

Samples of *Peperomia circinnata* Link var. *circinnata*. (*Piperaceae*). The essential oil of the Museu Paraense Emílio Goeldi is available from the authors.
